# Selenium concentrations in expressed human milk: a systematic review and meta-analysis

**DOI:** 10.1038/s41372-024-02057-4

**Published:** 2024-07-16

**Authors:** Ohad Ish Shalom, Ronit Lubetzky, Francis B. Mimouni, Dror Mandel

**Affiliations:** 1 Departments of Pediatrics and Neonatology, Dana Dwek Children’s Hospital, Tel Aviv Medical Center, Tel Aviv, Israel; 2 Faculty of Medicine, Tel Aviv University, Tel Aviv, Israel; 3 Leumit Health Services, Tel Aviv, Israel

**Keywords:** Paediatrics, Metabolism

## Abstract

**Background:**

We aimed to systematically review articles that reported selenium (Se) concentrations in human milk (HM).

**Methods:**

using PRISMA guidelines we searched MEDLINE and Embase using the terms “human milk and Selenium”. All clinical trials and systematic reviews were retrieved.

**Results:**

Out of 1141 articles, we reviewed 76 full-text articles, excluding 26. Thus, 50 articles were included in meta-analyses. Most studies (*N* = 31) did not specify whether milk was from mothers of preterm (PT) or fullterm (T) infants. In the other 19 studies, 1 examined PT-EHM, 15 T-EHM, and 3 both PT-EHM and T-EHM. In most studies, Se concentrations were highest in colostrum or until 3 months of lactation, then declined. Metaanalyzed means of PT-EHM or T-EHM provide estimates of Se intake a little higher than those reported to date, but still lower than current recommendations of intake.

**Conclusion:**

This study provides clinicians with useful estimates of Se intake in breast-fed infants.

## Introduction

Selenium is an essential nutrient and component of selenoenzymes such as active glutathione peroxidase (GSHPx) [1]. Since the latter enzyme plays a critical role in anti-oxidant defense, Se is important in the protection against oxidative tissue damage [1]. Selenium deficiency has been reported in children receiving long-term parenteral nutrition (PN) without Se supplementation and is associated with low plasma Se, erythrocyte macrocytosis, depigmentation, and muscle weakness [2]. Also, Se plays an important role in thyroid hormone production, as Se deficiency decreases the synthesis of thyroid hormones, by decreasing the function of selenoproteins, in particular iodothyronine deiodinases (DIOs), which are responsible for the conversion of T4–T3 [3]. In contrast, there seem to be little reports of Se toxicity in children [2], although symptomatic selenosis has been reported in children in Venezuela [4].

Selenium status might be more critical in preterm than in term infants [2]. Indeed, these infants are particularly prone to oxidative injury, for instance: bronchopulmonary dysplasia (BPD), retinopathy of prematurity, and cerebral white matter disease [2]. Low Se status has been documented in preterm infants and was found to be associated with BPD [5]. In addition, plasma Se concentrations have been found to decrease during the first weeks of life [6].

As stated by Finch, “trace mineral content in human milk is the “gold standard” for the healthy full-term infant who is breastfed” [7]. This is probably also valid for Se. The same author points out, however, that “for preterm infants, no gold standard exists” [7]. Thus, the knowledge of how much Se is present in human milk might help knowing how much Se is needed to prevent deficiencies, to meet in-utero accretion rates, and maybe also to avoid toxicity from excess intakes. Dietary Se is considered to be highly bioavailable with an intestinal absorption of up to 80% [8]. In previous reports, Se intake in breastfed infants has been estimated to be 2.3 µg/kg per day [8]. Darlow et al. performed a randomized, controlled, blinded trial of Se supplementation in 534 VLBW infants [9]. Selenium dose was 5 µg/kg/day enterally or 7 µg/kg/day parenterally. A significant effect was observed on Se plasma concentrations, which reached similar levels as had been reported in healthy-term infants [9]. The ESPGHAN guidelines on pediatric PN propose a parenteral intake of 7 µg/kg/day in preterm infants [2], similar to the dose given in the Darlow study [9], allowing to reach Se status similar to that of term infants [10]. In term infants and children, parenteral Se requirements are estimated to be 2–3 µg/kg/ day, based on enteral requirements and high bioavailability [7, 11].

We therefore conducted this systematic review to examine available evidence that documents Se concentrations in expressed human milk (EHM) and possibly provide a meta-analysis of this evidence. We also aimed to determine whether Se concentrations vary over time during lactation.

## Methods

### Search strategy

We conducted this systematic review up to April 17, 2023, and followed the PRISMA (Preferred Reporting Items for Systematic Reviews and Meta-Analyses) guidelines. We searched MEDLINE and Embase for relevant articles. A structured search strategy was constructed using high-frequency keywords and relying on thesaurus explode operation to maximize recall, then using search filters to achieve precision, as recently described [12]. We performed the searches in both MEDLINE and EMBASE using the terms “human milk and Selenium”. The results of the searches were filtered using document type (e.g., randomized controlled trial, systematic review, etc.) and English language. We then searched manually the reference lists of all selected papers to find additional references not found by the computerized search. All clinical trials and systematic reviews were included for full-text article review. Articles eligible for assessment were those involving measurements of Se concentrations in EHM. We excluded all articles that were not clinical trials or systematic reviews for full-text review, and we excluded as well all articles that did not provide the sample size, mean, and SD or SEM in a numerical manner. Our primary purpose was to review every single paper for new evidence related to Se concentrations in EHM.

### Data collection

One reviewer (OI) screened titles and abstracts of all records identified by the search and defined records as “order” or “exclude”. Then two investigators (FBM and OI) read all the full texts ordered to assess each article’s suitability for inclusion based on the pre-specified inclusion and exclusion criteria. Then the data were extracted independently using a data collection form to aid extraction of information on design, methods, and participants from each included study, and full texts of all relevant trials were obtained. Any disagreement was resolved through consensus between reviewers after thorough review of all articles.

### Statistical analyses

Meta-analyses were conducted in all the studies that reported Se concentrations in EHM. We also classified the data into the following: lactation days: 1 to 3 days (i.e., colostrum), 4 days–3 months, >3–6 months, >6–12 months, and >1 year. We noted for each study the biochemical method used. The Minitab Statistical Package, version 16 (Minitab; State College, PA) was used for analyses. Meta-analyses were calculated and expressed as weighted averages with pooled standard deviations, differentiating whether the samples analyzed were obtained from mothers of preterm infants (PT-EHM), term infants (T-EHM), or whether this information was not provided by the authors. Variation over time of Se concentrations was also studied using ANOVA with Bonferroni correction. Results are expressed as mean ± standard deviation, and a *P*-value of less than .05 was deemed significant.

## Results

**Fig. 1:**
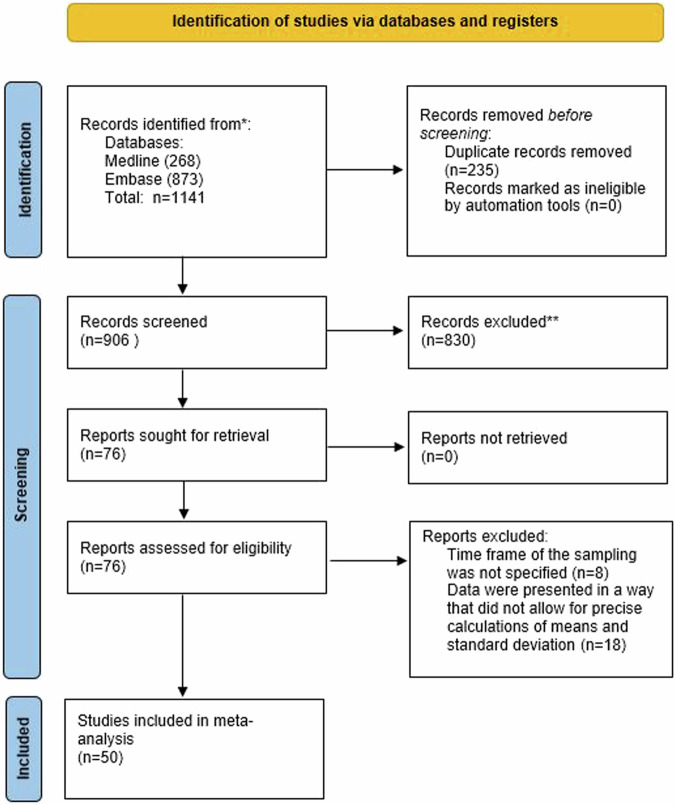
PRISMA 2020 flow diagram.

Figure 1 depicts a flow diagram of the literature search process. Briefly, out of 1141 articles initially retrieved by the search, we ended up reviewing a total of 76 full-text articles, of which 26 were excluded because either the time frame of sampling was not specified, or data were expressed in a way that did not allow for precise calculations of means and standard deviations. Thus, a total of 50 articles were included in the final meta-analyses. These articles are cited in an appendix to this article.

Table 1 depicts the metanalytic results (weighted averages with pooled standard deviations and sample size) of Se concentrations in EHM at the different periods of lactation described in the PT-EHM, T-EHM, and non-specified EHM (NS-EHM). Indeed, in 31 studies, results were described without any indication of whether the milk analyzed belonged to the mother of a preterm or a term infant. Importantly, not all studies analyzed Se human milk concentrations at all the periods of lactation that we defined. Therefore, the number of studies reported differs from period to period. Moreover, some studies reported concentrations at different periods during a specific time frame that we defined. For instance, in a given study, Se concentrations were analyzed in 2 subgroups at 1 and 2 months of lactation. In such a study, both subgroups of results were combined and included in the 0–3 months period. Most studies used atomic absorption spectrometry for Se measurements. As can be seen from the table, Selenium concentrations decreased from Colostrum values and first-trimester values over time (*P* < 0/001). Preterm milk Colostrum has a smaller but significantly different Se concentration than colostrum of term milk. Identifiable analyses for PT-EHM were conducted up to 3 months of lactation, showing a minimal, but statistically significant drop in mean Se values from 17.21 to 16.46 mcg/L. In T-EHM, follow-up was available until 7–12 months of age and showed a minor, but significant increase from colostrum to 3 months values, followed by a subsequent decline. The table also shows weighted values for studies that did not identify whether or not the milk was preterm or term. No statistics were performed comparing this group of studies with other ones because of the uncertain source of the milk samples.

**Table 1:** Meta-analyses of studies reporting mean selenium concentrations (µg/L) in human milk.

		Colostrum	>3 days–3 months	>3–6 months	>6–12 months	>12 months
Preterm	Number of studies	2	4			
	Combined sample size	60	346			
	Weighted average (µg/L)	17.21	16.46			
	Weighted SD (µg/L)	1.32	4.12			
Term	Number of studies	7	16	3	1	
	Combined sample size	202	1075	32	10	
	Weighted average (µg/L)	20.09	27.48	16.71	15	
	Weighted SD (µg/L)	6.53	5.23	3.76	1	
No class	Number of studies	13	28	11	3	1
	Combined sample size	545	4287	1199	17	32
	Weighted average (µg/L)	38.44	17.55	10.38	11.35	18.67
	Weighted SD (µg/L)	21.11	7.14	5.60	3.30	0.84

Statistical differences between mean selenium concentrations in human milk of different study subgroups:

CoPT(60) vs CoT(202) = *p* < 0.001.

CoPT(60) vs 0–3 mPT(346) = *p* < 0.001.

CoPT(60) vs CoNC(545) = *p* < 0.001.

CoT(202) vs 0–3mT(1075) = *p* < 0.0001.

PT: preterm, T: term, NC: no class, Co: colostrum, 0–3m: >3 days–3 months, in parentheses—combined sample size of each subgroup.

## Discussion

Our study provides with valuable data relating to pooled analysis, extracted from 50 studies of Se concentrations in EHM. It could be used for estimates of Se intake in human milk-fed infants during, and shortly after the first year of life.

We could not find in the literature similar studies which reviewed in a systematic manner Se contents of human milk over various periods of lactation.

Our study has, however, several inherent limitations. The first one is that our review, although systematic, is a cross-sectional analysis of the literature, and in no manner can be considered as longitudinal. Indeed, all 50 papers retained for analysis used different groups (in terms of stages of lactation). Some articles only measured Se concentrations in one specific period of lactation, while others used several periods, and mostly cross-sectionally. Consequently, at various periods of lactation, the pooled sample size varied from 27 combined measurements (in the > 6–12 months of lactation period) to 5708 measurements (in the ≥ 3 days–3 months of lactation). Obviously, the confidence interval of the measurement was affected by the sample size. Moreover, while Se values were apparently the highest in Colostrum and the first trimester of lactation, and subsequently increased, we believe that the temporal variations we describe here should be confirmed only in a longitudinal manner. Nevertheless, studies that were retained for analysis, whenever they had longitudinal measurements of colostrum and subsequent measurements, found universally that the colostrum concentrations were the highest ones (for instance references [13–15]). The reason for such high Se concentrations in colostrum might be the fact that Se is secreted together with proteins [16, 17], and we know that colostrum is characterized by a very high protein concentration [18].

Another limitation is that in we could not in this study correlate maternal Se status with EHM Se concentrations. A few individual studies that attempted such analyses had inconsistent results [19–21]. However, it is generally believed that human milk Se concentrations might be related to maternal intake, and therefore to geographic location of the population studied [17]. Unfortunately, it was not possible in this study, to precisely determine from the country of origin of each article what was the maternal Se intake.

From our results, we may calculate that an exclusively breastfed, 3 months old infant weighing an average of 6.4 kg, and taking an average of 700 cc of term human milk per day [22] is expected to have an average Selenium intake of 27.5 × 0.7 = 19.25 µg, or 3 µg /kg body weight. This number is actually higher than previous estimates of Se intake in breastfed infants of 2.3 mcg/kg per day [8]. It is however lower than current recommendations of Se intake, but these recommendations are not likely to lead to toxicity.

## Supplementary information

appendix 1

## Data Availability

The references of articles for metanalysis are presented in the appendix.
