# Supplementary material for: Selenium concentrations in expressed human milk: a systematic review and meta-analysis
Source: J Perinatol. 2024 Jul 16;44(11):1607–10. doi: 10.1038/s41372-024-02057-4 (PMC11518993; doi:10.1038/s41372-024-02057-4)
Supplement: Supplementary file 1 — appendix 1 [file 41372_2024_2057_MOESM1_ESM.docx]

References of articles for metanalysis

1. Torres MA, Verdoy J, Alegría A, Barberá R, Farré R, Lagarda MJ. Selenium contents of human milk and infant formulas in Spain. Sci Total Environ. 1999 Apr 5;228(2-3):185-92. doi: 10.1016/s0048-9697(99)00044-3. PMID: 10371053.
2. Butts CA, Hedderley DI, Herath TD, Paturi G, Glyn-Jones S, Wiens F, Stahl B, Gopal P. Human Milk Composition and Dietary Intakes of Breastfeeding Women of Different Ethnicity from the Manawatu-Wanganui Region of New Zealand. Nutrients. 2018 Sep 4;10(9):1231. doi: 10.3390/nu10091231. PMID: 30181524; PMCID: PMC6164561.
3. Smith AM, Picciano MF, Milner JA. Selenium intakes and status of human milk and formula fed infants. Am J Clin Nutr. 1982 Mar;35(3):521-6. doi: 10.1093/ajcn/35.3.521. PMID: 7064903.
4. Wei M, Deng Z, Liu B, Ye W, Fan Y, Liu R, Li J. Investigation of amino acids and minerals in Chinese breast milk. J Sci Food Agric. 2020 Aug;100(10):3920-3931. doi: 10.1002/jsfa.10434. Epub 2020 May 18. PMID: 32329067.
5. Hannan MA, Dogadkin NN, Ashur IA, Markus WM. Copper, selenium, and zinc concentrations in human milk during the first three weeks of lactation. Biol Trace Elem Res. 2005 Oct;107(1):11-20. doi: 10.1385/BTER:107:1:011. PMID: 16170218.
6. Kim ES, Kim JS, Cho KH, Lee KH, Tamari Y. Quantitation of taurine and selenium levels in human milk and estimated intake of taurine by breast-fed infants during the early periods of lactation. Adv Exp Med Biol. 1998;442:477-86. doi: 10.1007/978-1-4899-0117-0_57. PMID: 9635064.
7. Castriotta L, Rosolen V, Biggeri A, Ronfani L, Catelan D, Mariuz M, Bin M, Brumatti LV, Horvat M, Barbone F. The role of mercury, selenium and the Se-Hg antagonism on cognitive neurodevelopment: A 40-month follow-up of the Italian mother-child PHIME cohort. Int J Hyg Environ Health. 2020 Sep;230:113604. doi: 10.1016/j.ijheh.2020.113604. Epub 2020 Aug 29. PMID: 32871542.
8. Hannan MA, Faraji B, Tanguma J, Longoria N, Rodriguez RC. Maternal milk concentration of zinc, iron, selenium, and iodine and its relationship to dietary intakes. Biol Trace Elem Res. 2009 Jan;127(1):6-15. doi: 10.1007/s12011-008-8221-9. Epub 2008 Sep 19. PMID: 18802672.
9. Ellis L, Picciano MF, Smith AM, Hamosh M, Mehta NR. The impact of gestational length on human milk selenium concentration and glutathione peroxidase activity. Pediatr Res. 1990 Jan;27(1):32-5. doi: 10.1203/00006450-199001000-00007. PMID: 2296468.
10. Cumming FJ, Fardy JJ, Woodward DR. Selenium and human lactation in Australia: milk and blood selenium levels in lactating women, and selenium intakes of their breast-fed infants. Acta Paediatr. 1992 Apr;81(4):292-5. doi: 10.1111/j.1651-2227.1992.tb12228.x. PMID: 1606386.
11. Bratakos MS, Ioannou PV. Selenium in human milk and dietary selenium intake by Greeks. Sci Total Environ. 1991 Jun;105:101-7. doi: 10.1016/0048-9697(91)90332-9. PMID: 1925516.
12. Yanardağ R, Orak H. Selenium content of milk and milk products of Turkey. II. Biol Trace Elem Res. 1999 Apr;68(1):79-95. doi: 10.1007/BF02784398. PMID: 10208658.
13. Sabatier M, Garcia-Rodenas CL, Castro CA, Kastenmayer P, Vigo M, Dubascoux S, Andrey D, Nicolas M, Payot JR, Bordier V, Thakkar SK, Beauport L, Tolsa JF, Fumeaux CJF, Affolter M. Longitudinal Changes of Mineral Concentrations in Preterm and Term Human Milk from Lactating Swiss Women. Nutrients. 2019 Aug 9;11(8):1855. doi: 10.3390/nu11081855. PMID: 31405051; PMCID: PMC6723843.
14. Hojo Y. Sequential study on glutathione peroxidase and selenium contents of human milk. Sci Total Environ. 1986 Jun;52(1-2):83-91. doi: 10.1016/0048-9697(86)90106-3. PMID: 3726523.
15. Kantol M, Vartiainen T. Changes in selenium, zinc, copper and cadmium contents in human milk during the time when selenium has been supplemented to fertilizers in Finland. J Trace Elem Med Biol. 2001;15(1):11-7. doi: 10.1016/s0946-672x(01)80020-1. PMID: 11603821.
16. Li C, Solomons NW, Scott ME, Koski KG. Minerals and Trace Elements in Human Breast Milk Are Associated with Guatemalan Infant Anthropometric Outcomes within the First 6 Months. J Nutr. 2016 Oct;146(10):2067-2074. doi: 10.3945/jn.116.232223. Epub 2016 Aug 24. PMID: 27558578.
17. Mandiá N, Bermejo-Barrera P, Herbello P, López-Suárez O, Fraga JM, Fernández-Pérez C, Couce ML. Human Milk Concentrations of Minerals, Essential and Toxic Trace Elements and Association with Selective Medical, Social, Demographic and Environmental Factors. Nutrients. 2021 May 31;13(6):1885. doi: 10.3390/nu13061885. PMID: 34072740; PMCID: PMC8228089.
18. Tamari Y, Chayama K, Tsuji H. Longitudinal study on selenium content in human milk particularly during early lactation compared to that in infant formulas and cow's milk in Japan. J Trace Elem Med Biol. 1995 Mar;9(1):34-9. doi: 10.1016/S0946-672X(11)80006-4. PMID: 8846155.
19. Samuel TM, De Castro CA, Dubascoux S, Affolter M, Giuffrida F, Billeaud C, Picaud JC, Agosti M, Al-Jashi I, Pereira AB, Costeira MJ, Silva MG, Marchini G, Rakza T, Haaland K, Stiris T, Stoicescu SM, Martínez-Costa C, Vanpee M, Domellöf M, Euridice CG, Thakkar SK, Silva-Zolezzi I. Subclinical Mastitis in a European Multicenter Cohort: Prevalence, Impact on Human Milk (HM) Composition, and Association with Infant HM Intake and Growth. Nutrients. 2019 Dec 30;12(1):105. doi: 10.3390/nu12010105. PMID: 31905959; PMCID: PMC7019749.
20. Flax VL, Bentley ME, Combs GF Jr, Chasela CS, Kayira D, Tegha G, Kamwendo D, Daza EJ, Fokar A, Kourtis AP, Jamieson DJ, van der Horst CM, Adair LS. Plasma and breast-milk selenium in HIV-infected Malawian mothers are positively associated with infant selenium status but are not associated with maternal supplementation: results of the Breastfeeding, Antiretrovirals, and Nutrition study. Am J Clin Nutr. 2014 Apr;99(4):950-6. doi: 10.3945/ajcn.113.073833. Epub 2014 Feb 5. PMID: 24500152; PMCID: PMC3953887.
21. Wasowicz W, Gromadzinska J, Szram K, Rydzynski K, Cieslak J, Pietrzak Z. Selenium, zinc, and copper concentrations in the blood and milk of lactating women. Biol Trace Elem Res. 2001 Mar;79(3):221-33. doi: 10.1385/BTER:79:3:221. PMID: 11354347.
22. He MJ, Zhang SQ, Liu L, Han F, Chai Y, Zhang J, Wang S, Wang Q, Liu Y, Sun L, Lu J, Yang Q, Huang L, Huang ZW. Breast milk selenocystine as a biomarker for selenium intake in lactating women at differential geographical deficiency risk in China. Asia Pac J Clin Nutr. 2019;28(2):341-346. doi: 10.6133/apjcn.201906_28(2).0016. PMID: 31192563.
23. Li F, Rossipal E, Irgolic KJ. Determination of selenium in human milk by hydride cold-trapping atomic absorption spectrometry and calculation of daily selenium intake. J Agric Food Chem. 1999 Aug;47(8):3265-8. doi: 10.1021/jf990268d. PMID: 10552642.
24. Han F, Liu L, Lu J, Chai Y, Zhang J, Wang S, Sun L, Wang Q, Liu Y, He M, Mu W, Huang Z. Calculation of an Adequate Intake (AI) Value and Safe Range of Selenium (Se) for Chinese Infants 0-3 Months Old Based on Se Concentration in the Milk of Lactating Chinese Women with Optimal Se Intake. Biol Trace Elem Res. 2019 Apr;188(2):363-372. doi: 10.1007/s12011-018-1440-9. Epub 2018 Jul 16. PMID: 30014285.
25. Kim SY, Park JH, Kim EA, Lee-Kim YC. Longitudinal study on trace mineral compositions (selenium, zinc, copper, manganese) in Korean human preterm milk. J Korean Med Sci. 2012 May;27(5):532-6. doi: 10.3346/jkms.2012.27.5.532. Epub 2012 Apr 25. PMID: 22563219; PMCID: PMC3342545.
26. Zachara BA, Pilecki A. Selenium concentration in the milk of breast-feeding mothers and its geographic distribution. Environ Health Perspect. 2000 Nov;108(11):1043-6. doi: 10.1289/ehp.001081043. PMID: 11102294; PMCID: PMC1240160.
27. Funk MA, Hamlin L, Picciano MF, Prentice A, Milner JA. Milk selenium of rural African women: influence of maternal nutrition, parity, and length of lactation. Am J Clin Nutr. 1990 Feb;51(2):220-4. doi: 10.1093/ajcn/51.2.220. PMID: 2305708.
28. Zachara BA, Pilecki A. Daily selenium intake by breast-fed infants and the selenium concentration in the milk of lactating women in western Poland. Med Sci Monit. 2001 Sep-Oct;7(5):1002-4. PMID: 11535949.
29. Trafikowska U, Sobkowiak E, Butler JA, Whanger PD, Zachara BA. Organic and inorganic selenium supplementation to lactating mothers increase the blood and milk Se concentrations and Se intake by breast-fed infants. J Trace Elem Med Biol. 1998 Jul;12(2):77-85. doi: 10.1016/S0946-672X(98)80029-1. PMID: 9760415.
30. Ozdemir HS, Karadas F, Pappas AC, Cassey P, Oto G, Tuncer O. The selenium levels of mothers and their neonates using hair, breast milk, meconium, and maternal and umbilical cord blood in Van Basin. Biol Trace Elem Res. 2008 Jun;122(3):206-15. doi: 10.1007/s12011-008-8088-9. Epub 2008 Feb 27. PMID: 18301869.
31. Bianchi ML, Cruz A, Zanetti MA, Dorea JG. Dietary intake of selenium and its concentration in breast milk. Biol Trace Elem Res. 1999 Dec;70(3):273-7. doi: 10.1007/BF02783836. PMID: 10610066.
32. Ejezie FE, Okaka AC, Nwagha UI. Reduced maternal selenium levels in pregnant and lactating Nigerian women: should routine selenium supplementation be advocated? Niger J Med. 2012 Jan-Mar;21(1):98-102. PMID: 23301458.
33. Levander OA, Moser PB, Morris VC. Dietary selenium intake and selenium concentrations of plasma, erythrocytes, and breast milk in pregnant and postpartum lactating and nonlactating women. Am J Clin Nutr. 1987 Oct;46(4):694-8. doi: 10.1093/ajcn/46.4.694. PMID: 3661485.
34. Yamawaki N, Yamada M, Kan-no T, Kojima T, Kaneko T, Yonekubo A. Macronutrient, mineral and trace element composition of breast milk from Japanese women. J Trace Elem Med Biol. 2005;19(2-3):171-81. doi: 10.1016/j.jtemb.2005.05.001. Epub 2005 Oct 24. PMID: 16325533.
35. Loui A, Raab A, Braetter P, Obladen M, de Braetter VN. Selenium status in term and preterm infants during the first months of life. Eur J Clin Nutr. 2008 Mar;62(3):349-55. doi: 10.1038/sj.ejcn.1602715. Epub 2007 Mar 21. PMID: 17375120.
36. Li JZ, Yoshinaga J, Suzuki T, Abe M, Morita M. Mineral and trace element content of human transitory milk indentified with inductively coupled plasma atomic emission spectrometry. J Nutr Sci Vitaminol (Tokyo). 1990 Feb;36(1):65-74. doi: 10.3177/jnsv.36.65. PMID: 2362225.
37. Wälivaara R, Jansson L, Akesson B. Selenium content of breast milk sampled in 1978 and 1983 in Sweden. Acta Paediatr Scand. 1986 Mar;75(2):236-9. doi: 10.1111/j.1651-2227.1986.tb10191.x. PMID: 3962656.
38. Mandić Z, Mandić ML, Grgić J, Hasenay D, Grgić Z. Selenium content of breast milk. Z Lebensm Unters Forsch. 1995 Sep;201(3):209-12. doi: 10.1007/BF01192989. PMID: 7483855.
39. Dörner K, Schneider K, Sievers E, Schulz-Lell G, Oldigs HD, Schaub J. Selenium balances in young infants fed on breast milk and adapted cow's milk formula. J Trace Elem Electrolytes Health Dis. 1990 Mar;4(1):37-40. PMID: 2135956.
40. Wasowicz W, Gromadzinska J, Rydzynski K, Tomczak J. Selenium status of low-selenium area residents: Polish experience. Toxicol Lett. 2003 Jan 31;137(1-2):95-101. doi: 10.1016/s0378-4274(02)00383-1. PMID: 12505435.
41. Arias-Borrego A, Callejón-Leblic B, Rodríguez-Moro G, Velasco I, Gómez-Ariza JL, García-Barrera T. A novel HPLC column switching method coupled to ICP-MS/QTOF for the first determination of selenoprotein P (SELENOP) in human breast milk. Food Chem. 2020 Aug 15;321:126692. doi: 10.1016/j.foodchem.2020.126692. Epub 2020 Mar 26. PMID: 32251923.
42. Robberecht H, Roekens E, van Caillie-Bertrand M, Deelstra H, Clara R. Longitudinal study of the selenium content in human breast milk in Belgium. Acta Paediatr Scand. 1985 Mar;74(2):254-8. doi: 10.1111/j.1651-2227.1985.tb10960.x. PMID: 3993372.
43. Tamari Y, Kim ES. Longitudinal study of the dietary selenium intake of exclusively breast-fed infants during early lactation in Korea and Japan. J Trace Elem Med Biol. 1999 Nov;13(3):129-33. doi: 10.1016/S0946-672X(99)80002-9. PMID: 10612075.
44. Kumpulainen J, Vuori E, Kuitunen P, Mäkinen S, Kara R. Longitudinal study on the dietary selenium intake of exclusively breast-fed infants and their mothers in Finland. Int J Vitam Nutr Res. 1983;53(4):420-6. PMID: 6668143.
45. Kumpulainen J, Vuori E, Siimes MA. Effect of maternal dietary selenium intake on selenium levels in breast milk. Int J Vitam Nutr Res. 1984;54(2-3):251-5. PMID: 6500851.
46. Jariwala M, Suvarna S, Kiran Kumar G, Amin A, Udas AC. Study of the concentration of trace elements fe, zn, cu, se and their correlation in maternal serum, cord serum and colostrums. Indian J Clin Biochem. 2014 Apr;29(2):181-8. doi: 10.1007/s12291-013-0338-8. Epub 2013 May 21. PMID: 24757300; PMCID: PMC3990806.
47. Björklund KL, Vahter M, Palm B, Grandér M, Lignell S, Berglund M. Metals and trace element concentrations in breast milk of first time healthy mothers: a biological monitoring study. Environ Health. 2012 Dec 14;11:92. doi: 10.1186/1476-069X-11-92. PMID: 23241426; PMCID: PMC3599153.
48. Sziklai-László, I., Majchrzak, D., Elmadfa, I., & Cser, M. A. (2009). Selenium and vitamin E concentrations in human milk and formula milk from Hungary. *Journal of radioanalytical and nuclear chemistry*, *279*, 585-590.‏
49. Almeida AA, Lopes CM, Silva AM, Barrado E. Trace elements in human milk: correlation with blood levels, inter-element correlations and changes in concentration during the first month of lactation. J Trace Elem Med Biol. 2008;22(3):196-205. doi: 10.1016/j.jtemb.2008.03.007. Epub 2008 Jun 27. PMID: 18755395.
50. Brätter P, Brätter VE, Recknagel S, Brunetto R. Maternal selenium status influences the concentration and binding pattern of zinc in human milk. J Trace Elem Med Biol. 1997 Dec;11(4):203-9. doi: 10.1016/s0946-672x(97)80014-4. PMID: 9575470.
